# Cloacal Malformations: Technical Aspects of the Reconstruction and Factors Which Predict Surgical Complexity

**DOI:** 10.3389/fped.2019.00240

**Published:** 2019-06-14

**Authors:** Richard J. Wood, Carlos A. Reck-Burneo, Marc A. Levitt

**Affiliations:** ^1^Center for Colorectal and Pelvic Reconstruction at Nationwide Children's Hospital, Columbus, OH, United States; ^2^Department of Pediatric Surgery, Medical University of Vienna, Vienna, Austria

**Keywords:** cloaca, anorectal malformation, congenital, incontinence, surgical teaching

## Abstract

Cloacal malformations are rare anomalies which occur in one in 50,000 live births. Anatomically these anomalies are defined by the presence of a single perineal orifice. There is however a substantial range in their complexity. Defining these differences is key to surgical planning and timely referral of selected cases to centers with the capabilities to manage the most challenging cases. Traditionally the common channel length as measured during cysto-vaginoscopy has been used to differentiate between patients that can be repaired with a reproducible operation and those requiring a more complex repair. The quality and range of imaging available has advanced and thus a more detailed anatomic picture is now possible to help with pre-operative planning. Cross sectional imaging with 3D reconstruction has enhanced the understanding of the anatomic variations in these patients. In addition, the sacral ratio, previously thought to only have an influence on long term continence predictions, has been shown to not only forecast the presence of urological anomalies, but also the complexity of the malformation. In principle, cloacal malformations have two major components to the reconstruction. First, the rectum needs to be separated from the urogenital tract and second, the urogenital sinus needs to be managed to create a urethral orifice and vaginal introitus. The length of the urethra has been shown to be vital in deciding between the two main surgical maneuvers; a total urogenital mobilization (TUM) and a urogenital separation. The technical demands of a urogenital separation are significant and discussed here in detail. The need for vaginal replacement adds further complexity to the care of these patients and has also been shown to be related to the length of the urethra. Predicting complexity in an accurate and non-invasive way will facilitate the care of the most complex cloacal malformations and improve outcomes.

## Introduction

Cloacal malformations are characterized by a single perineal orifice and confluence, of the distal ends of the urological, genital, and gastrointestinal tracts and, represent the most complex end of the spectrum of female anorectal malformations. These rare malformations occur in 1 in 50,000 live births. Due to this very low incidence, the majority of pediatric general surgeons and urologists will be exposed to only a few cases through a career in practice, even in a busy center. This has led to the need to create protocols to allow for better management. The work of Pena and Hendren to establish treatment guidelines was an essential basis for the modern understanding of these malformations (1, 2). Repair of a cloaca separating a genital tract from urethra and emphasizing the urologic aspects was key foundational work (3). The establishment of the measurement of the common channel and the total urogenital mobilization (TUM) were both significant contributions to the field (4). Recently, as technology has allowed for more complex and accurate imaging techniques it has become clear that other aspects in the anatomical assessment are important for predicting complexity and surgical planning (5–7). The goal of pre-operative assessment is to predict in an accurate manner which cases of cloaca can be repaired with a reproducible operation, the TUM, and which cases require a more complex repair (urogenital separation) with or without the added complexity of vaginal replacement (8).

## Factors in the Initial Assessment Which Affect Treatment

The most important aspect in the care of a patient with a cloacal malformation is making an accurate initial diagnosis. This can be made clinically with good lighting and an effective technique. Initially the perineum is spread to identify the fact that there is no anal opening. Then the labia are lifted up and out to reveal a single perineal orifice. After the diagnosis is made the most important assessment is whether there is significant hydrocolpos present, causing hydronephrosis, by performing early pelvic and renal ultrasound, and if so, whether it is amenable to perineal drainage. This decision making can be challenging for several reasons. First, at this early stage patients may be in the physiologic oliguric stage and may therefore, not produce enough urine to cause significant hydronephrosis despite it being present *in utero*. Second, almost all patients with a cloacal malformation require a colostomy as part of their initial treatment. The ideal time to drain the hydrocolpos would be at the time of the colostomy formation. However, it may not yet be apparent whether intermittent perineal catheterization (a viable option to manage hydrocolpos), is working by the time the patient undergoes colostomy formation. In the past this has led surgeons to drain the hydrocolpos formally with a vaginostomy, however there is no good evidence that drainage with a vaginostomy is superior to intermittent catheterization (9–11), and therefore, there has been a shift in practice in many centers away from default vaginostomy formation. Whichever, drainage method is chosen, there needs to be a commitment to ongoing care. Regular ultrasound investigations to confirm decompression of the hydrocolpos and improvement of the hydronephrosis are essential to prove that the method chosen is working.

Much speculation exists about the ideal diversion site for the colon in cloaca patients. There are variations in practice but the principles remain to create a completely diverting stoma which can be successfully managed and is created in an adequate position in the bowel which does not interfere with the future rectal pull through and the arcades which may be needed for a future vaginal replacement (12). We have formed end stomas in the descending sigmoid junction with a mucus fistula at a separate site and have found these to be successful, however other options do exist. The disadvantage of transverse colostomies are; increased urine absorption which may lead to acidosis, increased rates of prolapse, and difficulty distending the rectum for imaging of the distal colon for surgical planning. Several authors propose the use of loop stomas in the literature and if these can be completely diverting, which requires a refined technique, then the evidence would suggest they are a reasonable alternative (13).

### Imaging Options Prior to Definitive Care

A detailed understanding of the anatomy of a cloacal malformation is critical to the successful repair of these challenging surgical patients. There are multiple components to consider and having an organized approach is beneficial. The use of multi-modal and multi-disciplinary input has been found to provide all the necessary information to make good decisions (5). All patients should undergo an endoscopic examination just prior to reconstruction. We do not advocate endoscopy in the neonatal period as this can cause trauma to delicate structures, and the images provided by small endoscopes is often suboptimal.

**Figure 1:**
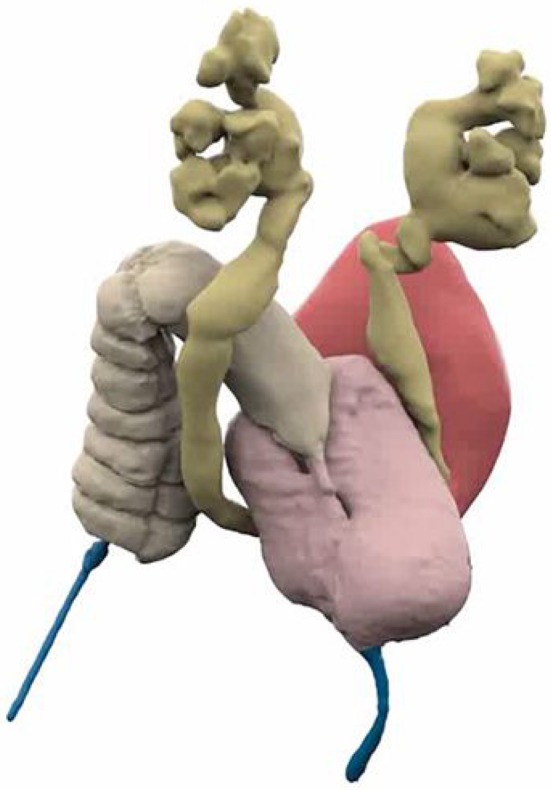
3D Reconstructed Cloacagram with bladder marked in magenta, vaginas in pink, bowel in tan, and common channel in blue. Reproduced with permission from © Center for Colorectal and Pelvic Reconstruction at Nationwide Childrens Hospital.

Endoscopy should be performed with all surgical teams (colorectal, urology, and gynecology as available) and radiology present. A detailed understanding of the anatomy of the urogenital tracts and the location of the rectal fistula can be obtained. The bladder and ureteric anatomy can be reviewed as well as the anatomic characteristics of the bladder neck and the urethra above the common channel. Measurements of the length of the urethra and the length of the common channel can be taken however the measurements taken with 3D reconstructed imaging are more accurate than those taken with endoscopy (Figure 1). This relates to the angle change of the common channel and urethra as they traverse the area posterior to the pubic symphysis and is therefore, more dramatic in longer common channel malformations. In addition, endoscopy is able to delineate the anatomy of the female genital tract if it is connected to the common channel. The presence of a longitudinal vaginal septum and uterine didelphys can be diagnosed as well as the number of cervices and their patency. The presence of a didelphys configuration is the most definitive from a diagnostic point of view, the presence of a single cervix could mean either normal uterine anatomy or perhaps a bicornuate uterus which may not be obvious on imaging. Multi-modal imaging and longitudinal follow up is required, especially around puberty, to fully define the uterine anatomy in many cases (14). In addition, the location of the rectal fistula can be assessed during endoscopy, however, the location is only part of the story. It is important to not only know the location of the fistula's entry into the vagina or common channel but also the location of the true rectum in the pelvis. Spatial understanding of the relationships between all structures requiring reconstruction and the pelvis is vital.

What imaging techniques are available and how should they be used?

The options for cloacal assessment are 2D fluoroscopy, 3D reconstructed fluoroscopy with or without the ability to manipulate the imaging, 3D printed images, and pelvic MRI with or without MR urography. When analyzing imaging options it is important to assess what information the different modalities are able to provide and additionally how easily this can be interpreted by surgical teams. The necessary measurements would appear to be obtainable from any of these modalities however their interpretation differs between the different modalities. Levels of experience definitely affects the ability to understand these images with less experienced surgeons faring better on more complex imaging, approaching the abilities of experienced surgeons (15). The fact that cloacal malformations are rare further emphasizes the need to use more complex imaging modalities. While 3D printing may be beneficial for the purposes of explanation to families (16), it adds significant cost and does not add significantly to the understanding of the anatomy by the surgical team (15). MRI is able show great soft tissue definition which may further aid the spatial understanding of the anatomy of pelvic musculature and hollow visceral structures (17). There may be questions about the ability of MR to define fine structures like the common channel and urethral length accurately and at this stage should probably be used in conjunction with fluoroscopy. Other modalities like contrast enhanced ultrasound are currently under investigation and may hold future promise. At the conclusion of the endoscopy and review of the imaging, the surgical team should know; (1) the length of the common channel, (2) the length of the urethra, (3) the anatomy of the vagina or vaginas, and sometimes the anatomy of the upper genital tract, and (4) the location of the rectal fistula and the true rectum and its position in the pelvis, notably the pubo-coccygeal (PC) line. This information will allow the surgical team to decide on a surgical strategy which we have found to be consistently predict the correct surgical plan (8).

### Surgical Options for Definitive Reconstruction

The role of pre-operative imaging and endoscopy is to determine which options for reconstruction will be most effective in achieving the best functional outcome for the patient. For practical purposes the malformation can be divided into an anterior and posterior compartment. The posterior compartment refers to the rectum, which in all cases needs to be separated from the urogenital tracts and mobilized into position as defined by the muscle complex (vertical fibers) of the anal sphincter mechanism. The PC line is a good guide as to whether the true rectum can be mobilized from a posterior sagittal approach or will require a trans-abdominal approach. If the normal lumen of the rectum lies below the PC line it can reliably be mobilized through a posterior sagittal incision. If above the PC line, an abdominal component will be needed, either via laparotomy or laparoscopy. When considering the rectal portion of the repair, consideration should also be given to the length of colon from the mucus fistula and options for vaginal replacement, should one be necessary.

The anterior compartment contains the urogenital tracts and can be seen as a separate but related structure. Recent work has helped to define the length of the female urethra as a marker of the location of the bladder neck in the female pelvis and its relationship with the structures of the pelvic floor. These data indicate that a minimum urethral length for a normal female between age 6 and 36 months is 1.5 cm and the mean length is 2.5 cm with minimal growth occurring during this period (18). Embryologically it would be difficult to understand the bladder neck forming below the urogenital diaphragm in a normal, asymptomatic female. By inference, then the minimum length for the urethra after reconstruction of a cloacal malformation should be 1.5 cm. This concept greatly simplified the decision making in urogenital reconstruction (8). After endoscopy and cloacagram, it should be clear what the length of the common channel and urethra (above the common channel) are. If the common channel is <3 cm in length and the urethra is at least 1.5 cm in length then the patient should be reconstructed with a TUM. In patients suitable for TUM who have a rectum above the PC line the rectum can be mobilized laparoscopically prior to performing the TUM. The laparoscopic portion of this procedure very closely resembles the laparoscopic mobilization of the rectum in the male recto-bladder neck fistula. If the fistula reaches low down on the posterior wall of the vagina/s then it does not need to be divided as this can easily achieved during the posterior sagittal portion of the procedure and brought down. If however, the rectal fistula implants high then it should be divided in the same way as is done in the laparoscopic assisted anorectoplasty (LAARP) prior to starting the TUM.

If the common channel is >3 cm in length or the urethra is <1.5 cm then the TUM is not appropriate for reconstruction. While the TUM has many advantages, amongst others, reproducibility, and the prevention of urethra-vaginal fistulas (4), its indications should not be stretched. This will prevent excessive mobilization of the bladder and leaving patients with overly short urethras and therefore, bladder necks below the urogenital diaphragm. In patients who do not qualify for a TUM, a urogenital separation will be required. This is a major technical change from previously reported recommendations, which recommend a trans-abdominal TUM (2), a procedure we believe should almost never be performed. Technically this is a demanding maneuver and the risks of urethro-vaginal fistula are not insignificant, however from a functional point of view it does appear to be necessary. Minimizing the risk of urethro-vaginal fistula is vital and involve a number of key technical steps (8) which will be described. The vagina/s are mobilized off of the common channel and urethra, much like the rectum is mobilized in the male recto-urethral fistula. There are a number of similarities, notably the lower the vagina reaches into the pelvis, the more common wall dissection there is and the more challenging is the separation. The genital tract tends to envelop the posterior urethra and bladder neck, and this is why a clear spatial understanding, from the cloacagram, is so important. Once the decision is made that a urogenital separation is required, the next step is decide whether this process can be achieved posterior sagittally only, or whether it will require an additional abdominal approach. The abdominal portion can be performed with minimally invasive techniques (laparoscopic or robotic) or by laparotomy. The goal of this portion is the creation of a smooth, easily catheterizable urethra. Care must be taken not to narrow the common channel and for this reason it is usually advantageous to start the separation from the posterior approach, unless the urethra-vaginal fistula lies well-above the PC line.

The final part of the pre-operative decision making is whether a vaginal replacement is needed and if so, what tissue to use for this purpose. The options are ileum, colon, and rectum. The sigmoid colon has a thinner pedicle and may be technically easier to mobilize to the perineum and this is usually our first option. Small bowel is often a good option, but the pedicles can be delicate during extensive mobilization. The best colonic segment, we find, is often the functional stoma (i.e., the left colon) which makes pre-operative bowel preparation essential. We believe the vaginal switch should no longer be performed, given the high ischemia rate and the better options available (19). There are no data yet, to indicate the benefit of one technique over another. The rectum is an option in highly selected cases where the patient's potential for bowel control is very poor (i.e., Myelomeningocele, very long common channel, or absent sacrum). However, if the rectum is used that may have an impact on fecal continence.

In the future we hope that tissue engineered vagina (20) becomes available at this point in the operation and confines vaginal replacement with bowel to the history books.

The strategy described above has been used successfully in the repair of 84 primary cases of cloaca so far without having to change the surgical plan. In each case a successful repair was performed. There are no instances of the urethra not reaching during TUM which thus avoided a subsequent urogenital separation, a situation which can lead to urethral loss. Based on this experience we propose three clear strategies (8):

1. Type 1 cloaca: common channel <1 cm in length: The urethra is left untouched and the surgical team performs an introitoplasty and a posterior sagittal anorectoplasty (PSARP).
2. Common channel <3 cm in length and a urethral length of at least 1.5 cm: TUM and PSARP which may require laparoscopy or open approach if the rectum is high.
3. Common channel >3 cm or urethra <1.5 cm: Urogenital separation with common channel kept as urethra and PSARP. A proportion of these patients require vaginal replacement with colon, rectum or small bowel. Open or MIS techniques may be required in these cases, especially where rectum or urogenital confluence lies above the PC line.

## Technical Aspects

**Figure 2:**
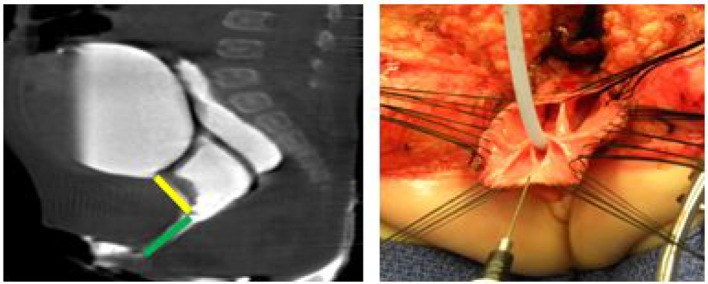
A 2D Cloagagram with the urethra marked in yellow and the common channel marked with green. This demonstrates a common channel of 2.5 cm and a urethra of 2 cm. The operative photo shows the splitting of the common channel during the Total Urogenital Mobilization (TUM), leaving only the urethra (green) to be sutured to the perineum. Reproduced with permission from © Center for Colorectal and Pelvic Reconstruction at Nationwide Childrens Hospital.

**Figure 3:**
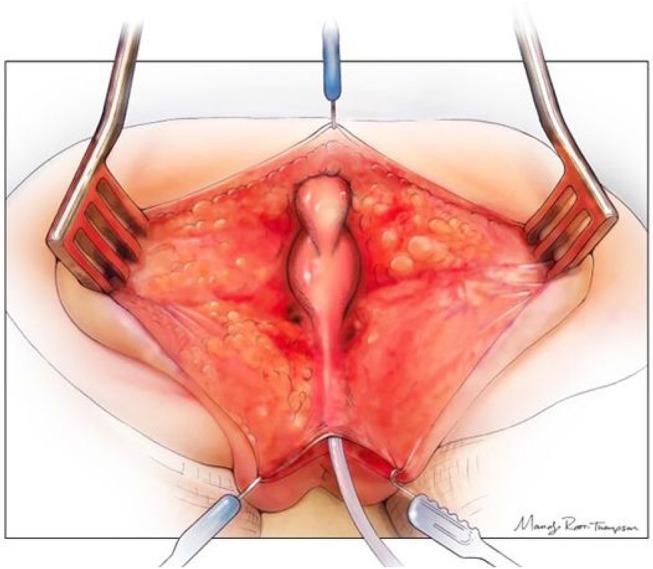
The posterior sagittal view with the rectum, vagina, and common channel dissected out but not opened. Reproduced with permission from © Center for Colorectal and Pelvic Reconstruction at Nationwide Childrens Hospital.

**Figure 4:**
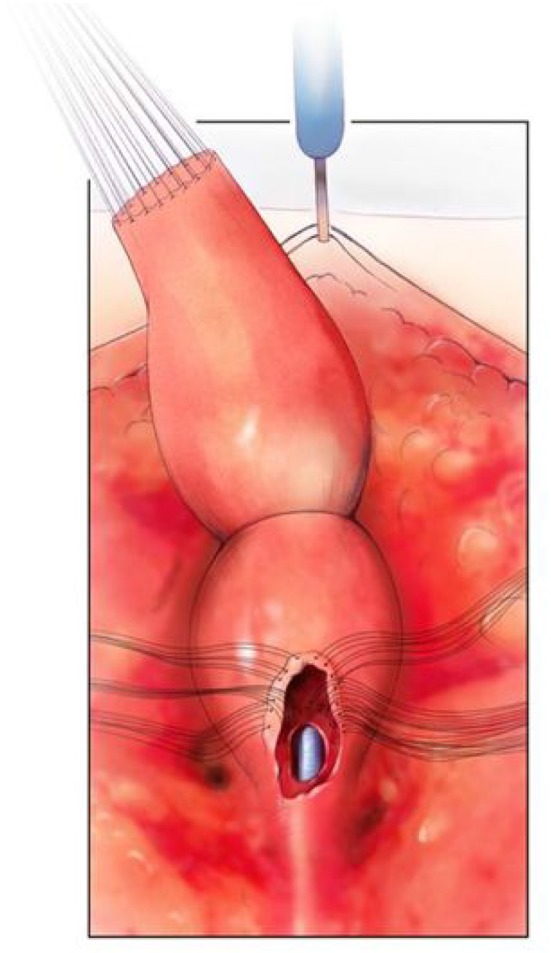
The rectum mobilized free from the vagina and the vagina opened. There are sutures on the edge of the vagina above the urethro-vaginal fistula in preparation for separation. The urethral catheter is visible. Reproduced with permission from © Center for Colorectal and Pelvic Reconstruction at Nationwide Childrens Hospital.

**Figure 5:**
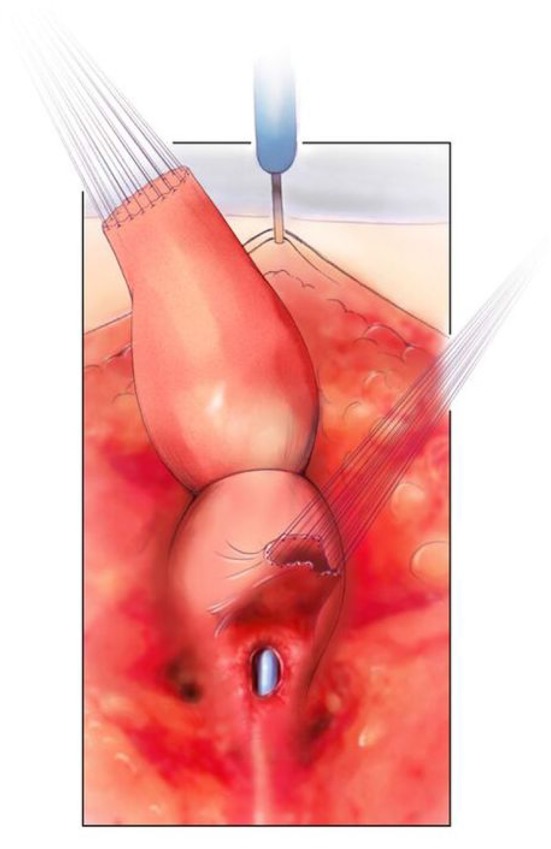
The rectum is mobilized and the vagina is being mobilized off the common channel, urethra, and bladder neck. A urethral catheter visible. Reproduced with permission from © Center for Colorectal and Pelvic Reconstruction at Nationwide Childrens Hospital.

1. In a type 1 cloaca the patient is placed in prone position and the anal sphincter complex is marked with an electrical stimulator. An incision is then carried from the posterior extent of the muscle complex (sphincter) through to the common channel, opening the common channel to reveal the urethral take-off, rectum, and vagina/s. The rectum is then mobilized, taking care to protect both the intra-mural blood supply of the rectum and the posterior vaginal wall. Once the rectum is separated, the vagina is opened to facilitate the formation of an adequate introitus. An electrical stimulator is employed to mark the anterior and posterior extent of the muscle complex. The length of perineal body, between 1 and 2 cm depending on the age and size of the patient is then selected. The remainder of the incision is left for the introitus. It is important that the repair is planned in this fashion to allow the rectum to be placed in the muscle complex and to create an adequate perineal body. The perineal body is repaired in layers with 3–0 long term absorbable interrupted sutures and the skin of the perineal body is repaired with vertical mattress sutures to take tension off the skin edge. The PSARP and posterior sagittal incision are repaired in the standard fashion and a vaginal septum, if present, is divided at the time of the introitoplasty.
2. The description of the technique of the TUM has been previously reported and the technique we employ does not differ from this approach (4). The surgery is started with mapping of the anal sphincter. In this instance a full posterior sagittal incision will be required. The incision is carried from the coccyx to the common channel and the common channel is opened posteriorly until the rectum and vagina/s are visible. The rectum is then mobilized in the same way as described above. Thereafter sutures are placed in the edge of the urogenital complex and the entire structure is mobilized in a full thickness fashion without compromising the integrity of the wall (Figure 2). The tissue is divided 5 mm posterior to the clitoris to allow for the urethroplasty to be placed directly posterior to the clitoris in a visible position in case intermittent catheterization is required and to avoid vaginal voiding. The posterior lateral blood supply of the vagina may need to be ligated to allow the vagina to be adequately mobilized. The careful division of the suspensory ligaments of the urethra will be needed to allow for mobilization of the urethra into the position posterior to the clitoris. There is much discussion in the literature regarding partial and complete TUM. The reality is that only those fibers needed to adequately mobilize the urogenital complex should be divided and this may vary from case to case. Once all three structures (rectum, vagina, and urethra) are adequately mobilized to reach the perineum the reconstruction proceeds in the standard fashion. After the anterior common channel has been divided and the urethra has been adequately reconstructed, the sphincter complex, and perineal body are planned and the remaining incision is used to create the introitus. The split common channel can be used to form the labia minora on each side. As above, a longitudinal vaginal septum if present can be divided at the time of the introitoplasty.
3. Patients requiring a urogenital separation require more challenging reconstructive techniques. Identifying these patients up front may facilitate referral to high volume centers as required. Except in cases of very long common channels (with all three structures above the PC line), we would advocate starting with a posterior sagittal approach. The incision runs from the coccyx to just posterior to the common channel. Where possible the common channel should be left intact at the perineal level (Figure 3). The wound is widely opened and the surgeon's understanding of whether the rectum and vagina/s lie above or below the levators (PC line) is important. If present in the posterior sagittal field the rectum should identified and mobilized as previously described above. The rectal attachment to the vagina/s or common channel needs to be identified, confirming what was seen on preoperative imaging, and divided. If the connection is to the common channel, care must be taken not to injure or narrow the common channel. At this stage the posterior vagina is opened close to where it joins the common channel (urethro-vaginal fistula). Sutures are placed on the edges of the vagina/s and the surgeon is able to look inside and identify the connection between the vagina/s and the common channel and urethra (Figure 4). The next stage is to start the separation of the vagina/s from the common channel, urethra, and bladder neck. This is done in the same way as is performed in a male undergoing a PSARP for a recto-urethral fistula, with lateral dissection done first, then anterior (Figure 5). Once the bladder neck is reached or the dissection becomes too high, the surgery should be continued in a trans-abdominal fashion. This will prevent placing the ureters in danger of being injured. If the ureters are ectopic they can be stented cystoscopically at the start of the procedure.

**Figure 6:**
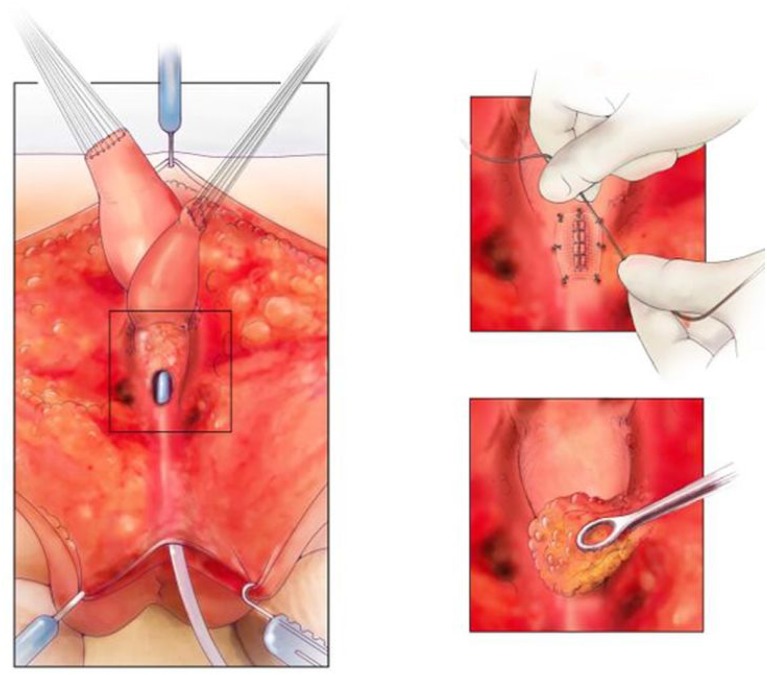
On the left, is the fully separated rectum, vagina, and urological tract with common channel kept as urethra. The top right picture shows the urethra repaired and the repair covered with SIS. The bottom right picture shows the repair being covered with an ischiorectal fat pad. Reproduced with permission from © Center for Colorectal and Pelvic Reconstruction at Nationwide Childrens Hospital.

The common channel needs to be meticulously repaired in order to leave the patient with a catheterizable channel. Urologic involvement can be advantageous and a repair in multiple layers with 5–0 PDS is our preference (Figure 6). Meticulous technique is essential and on table flexible cystoscopy can be helpful in some cases to identify precisely where the repair of the common channel is needed. Thereafter, the repair is covered with a single layer of SIS and the previously described ischiorectal fat pad (21). This repair is vital to the successful reconstruction of these patients (8), in an attempt to avoid a urethro-vaginal fistula. Using this technique and adequate bladder drainage for at least 1 month has led to a fistula rate of <5% (2/41) in patients requiring separation. This is less than reported but still challenging to manage. Bladder drainage can be accomplished with a transurethral Foley catheter or circle stent if the patient has a vesicostomy. The advantage of the circle stent is that there is no balloon in the bladder which may help to prevent bladder spasms in the post-operative period.

The abdominal portion of the procedure can be accomplished with open or MIS techniques. From a trans-abdominal approach the ureters are carefully identified and protected and the separation is continued in the midline until the structures are fully separated. The vagina/s are the mobilized until they reach the perineum, preserving both round ligaments, and thus their main blood supply. Advanced technology like SPY can be used to assess blood supply during this process (material accepted for presentation at the American Pediatric Surgical Association, May 2019 meeting, but not yet published). At this stage either the vagina/s are able to reach the perineum or a tissue replacement will be required to bridge the gap.

If vaginal replacement is required; rectum, colon, and ileum are available. Rectum should only be considered in cases where the likelihood of fecal continence is low due to a sacral ratio of <0.4 or very abnormal spinal development. After the tissue for replacement has been prepared and is determined to reach the perineum with adequate blood supply, and no tension, it can be anastomosed to the distal native vagina/s. On occasions the blood supply dictates that the neo-vagina be placed in an anti-peristaltic direction. If there is a longitudinal vaginal septum this may be divided to allow for future menstrual egress. The neo-vagina is then carefully pulled through to the perineum and reconstructed posterior to the common channel opening which has become the urethra. The posterior extent of the labia majora is often a helpful guide for the location of the posterior limit of the introitus. As before, the location of the rectum and perineal body should be planned prior to beginning the introitoplasty.

The patient should undergo standard post-operative care, but we would advocate for an examination under anesthesia, cystoscopy, and vaginoscopy between 4 and 6 weeks post-operatively to carefully examine for good healing and identify any post-operative complications. At this point assessment for intermittent catheterization and removal of foley catheter or circle stent is performed. A suprapubic tube or vesicostomy can be left in place until the ability to catheterize the urethra and adequately drain the bladder, if necessary, is ascertained.

### Long Term Follow up

Long term follow up needs to include three key components:

1. Bowel management strategies to optimize fecal continence when possible and otherwise social cleanliness strategies. This can be accomplished with the use of laxatives and/or antegrade or retrograde enemas.
2. Urological follow up with the main emphasis on renal protection needs to be provided. This includes specialty follow up with renal function testing, regular ultrasounds of the bladder and kidneys and urodynamic assessments.
3. Gynecological follow up is paramount to educate the patient about the special needs of this population and to assure unobstructed Mullerian anatomy. The exact anatomical understanding of the Mullerian system can be challenging at the time of the original repair, therefore we would advocate for Mullerian imaging starting with a pelvic ultrasound at 6 months after thelarche to diagnose an obstructed system (14).

## Outcomes

What post-operative outcomes are important? Understanding what is important to patients and families is vital to understanding which outcomes to assess and attempt to improve. Two components need to be addressed in this patient population. The initial results in the form of quality data all should refer to initial post-operative results; stricture rates of anoplasty and introitus, tissue or graft loss, wound dehiscence, need for reoperation, and ability to catheterize the urethral channel. Thereafter functional outcomes need to be reviewed, e.g., fecal and urinary continence, need for urological reconstruction, the ability to pass menses without pain or obstruction and successful sexual outcomes. These functional outcomes do not occur at the same time, stressing the need for a commitment for ongoing care and a well-defined care pathway. The new approach to consider urethral length in surgical planning developed from the observation that 75% of patients in two large published series were either suffering from urinary incontinence or were unable to void spontaneously (2, 22). In time we plan to report the functional outcomes of this group of patients but so far results are encouraging. It appears that a consequence of increased urogenital separation is an increased need for vaginal replacement. There are no good data on sexual and obstetric outcomes after vaginal replacement and this is something which needs to be addressed in a patient centered care model (23). Indeed even after cloacal reconstruction the exact gynecological anatomy was only known in 86% of patients in a tertiary referral center (14). This again stresses the need for longitudinal follow up. Much emphasis is placed on the pursuit of fecal and urinary continence and rightly so, however, 20–50% of cloaca patients will experience renal impairment during their lifetime and this should always be kept in mind. Measures aimed at aiding urinary continence may have unintended consequences on renal function and need to be carefully considered. Better bladder management to ensure good emptying is vital. Longitudinal patient and caregiver reported outcomes (PROMS) and experience measures (PREMS) need to form part of the care of these complex patients as well as a greater understanding of what life is like for adolescent and adult patients to help inform selection of reconstructive options.

## Author Contributions

RW: main author. CR-B: author, critical review, and data analysis. ML: senior reviewer and author.

### Conflict of Interest Statement

The authors declare that the research was conducted in the absence of any commercial or financial relationships that could be construed as a potential conflict of interest. The reviewer RR declared a past co-authorship with one of the authors ML to the handling Editor.
